# Sustainable dual-drug analysis: a synchronous spectrofluorimetric approach with integrated greenness and whiteness metrics for favipiravir and levofloxacin

**DOI:** 10.1038/s41598-026-35670-8

**Published:** 2026-02-03

**Authors:** Hany A. Batakoushy, Asmaa Othman El-Demerdash, Ashraf M. Taha, Ola M. Abdallah, Asmaa Abbas Nazer

**Affiliations:** 1 Department of Pharmaceutical Analytical Chemistry, Faculty of Pharmacy, Menoufia University, Shebin Elkom, 32511 Egypt; 2 Department of Pharmaceutical Analytical Chemistry, Faculty of Pharmacy, Menoufia National University, 70th km Cairo-Alexandria Agricultural Road, Menoufia, Egypt; 3 Pharmaceutical Analytical Chemistry, Faculty of Pharmacy (Girls), Al-Azhar University, Cairo, Egypt; 4 B.Sc. of Pharmaceutical Sciences, Faculty of Pharmacy, Sinai University, Al-Arish, Egypt

**Keywords:** Synchronous, Spectrofluorimetry, Favipiravir, levofloxacin, Whiteness, Greenness, Chemistry, Drug discovery

## Abstract

Innovative and sustainable synchronous spectrofluorimetric techniques were developed for the simultaneous assessment of favipiravir (FVP) and levofloxacin (LEV). Some COVID-19 treatment plans call for these drugs to be given together, so reliable approaches are needed to confirm that the studied drugs are of good quality. FVP and LEV are determined with high sensitivity in the nanogram per milliliter range, with high accuracy (recoveries of 99.34% ± 1.10 and 98.87% ± 0.44, respectively). Method validation was conducted in accordance with ICH guidelines. Moreover, several metric systems (Analytical Eco-Scale, GAPI, AGREE) are used to assess environmental sustainability, which confirms the greenness of the proposed method. Also, blueness and whiteness criteria (BAGI, RGB 12) were used to check the usefulness and analytical effectiveness of the established method. As this spectrofluorimetric method is simple, rapid, and inexpensive, it is preferred over other analytical techniques for the routine analysis of the drug combination in bulk powders and pharmaceutical formulations.

**Supplementary Information:**

The online version contains supplementary material available at 10.1038/s41598-026-35670-8.

## Introduction

In December 2019, China notified the World Health Organization about several cases of pneumonia of unknown etiology, later diagnosed as coronavirus disease (COVID-19)^1^. The rapid global spread of the pandemic accelerated research into antiviral drugs. One crucial strategy was to use FDA-approved drugs already on the market^2–4^. Favipiravir (FVP) emerged as a promising antiviral due to its ability to inhibit RNA polymerase^5,6^. Concurrently, antibiotics such as Levofloxacin (LEV) were widely used to control secondary bacterial infections in severe cases^7–9^. This therapeutic approach created a critical demand for robust analytical methods to support drug development, quality control, and pharmacokinetic studies. Both FVP (C_5_H_4_FN_3_O_2_) and LEV (C_18_H_20_FN_3_O_4_) are essential to this pharmaceutical concern (Fig. S1)^10^. Consequently, this study focuses on the development and validation of a novel, reliable analytical technique for the simultaneous determination of FVP and LEV to meet a critical requirement for the continuous enhancement of COVID-19 treatment protocols. Currently, no established technique exists for the concurrent assessment of FVP and LEV in pure and co-formulated pharmaceutical preparations. The methods reported for the quantitative determination of both drugs include liquid chromatography^1,11–22^, UV-spectrophotometry^23–30^, spectrofluorimetry^1,31–39^, and TLC-densitometry^40–46^.

The proposed spectrofluorimetric approach offers straightforward processing, high time efficiency, and cost-effectiveness, making it more advantageous than other analytical techniques^47–49^. TLC-densitometry is a rapid qualitative assessment; however, it lacks precision and resolution. A significant drawback of HPLC technology is its high cost, driven by the need for expensive equipment, specialized materials, and installation. Also, HPLC requires specialized, experienced staff, which is a time-intensive process^50,51^. Spectrophotometric^23–30^ and electrochemical^52^ methods are inadequate for identifying specific analytes and exhibit limited detection ranges. Furthermore, electrode fouling, selectivity challenges, and regulatory limitations represent constraints in electrochemical analysis^53^. Conversely, the established spectrofluorimetric approach stands out for its time and cost savings, enhanced sensitivity, and selectivity^54–56^. Additionally, simplicity and efficiency are afforded by the ease of use of the synchronous mode, which facilitates optimal band resolution^57^.

The proposed method aims to showcase green, white, low-energy devices, along with assessments of high sensitivity, selectivity, cost-effectiveness, and quantum efficiency, using a synchronous spectrofluorimetric method to achieve potential band resolution with effective spectral overlaps.

## Experimental

### Apparatus

The following instrument was used to perform the excitation and emission fluorimetric measurements: an Edinburgh, UK-based FS5 spectrofluorimeter equipped with a 150 W xenon lamp, a 1-cm quartz cell, and connected to Fluoracle® software. The slit widths were adjusted to 2 nm. pH correction was performed using a Jenway 3520 pH meter and a glass pH electrode (Jenway, Staffordshire, United Kingdom).

### Materials and reagents

Levofloxacin (99.75%) was graciously offered by Epico Pharmaceutical Company, 10th of Ramadan City, Egypt. Favipiravir (99.80%) was kindly supplied by Eva Pharma Company, Mashaal, Giza, Egypt. Avipiravir® Tablets (claimed labeled amount: 200 mg Favipiravir per tablet) were manufactured by Eva Pharma Company. Tavanic® Tablets (claimed labeled amount: 500 mg levofloxacin per tablet) manufactured by Sanofi Aventis Company. Ethanol, Methanol, and Acetonitrile were purchased from Piochem Chemicals Company, Al-Qasr Al-Ayni, Egypt. Boric acid, hydrochloric acid, and sodium hydroxide were purchased from EL-Nasr Company, Giza, Egypt.

### Standard solutions preparation

Standard stock solutions of FVP and LEV were freshly prepared at a concentration of 0.1 mg mL^−1^ by dissolving 10.0 mg of each compound in distilled water, and the final volume was adjusted to 100 mL in a volumetric flask. Then, working standard solutions for each pharmaceutical were prepared by diluting with distilled water to yield a concentration of 1 μg mL^−1^.

### Procedures

#### Construction of calibration graphs

Various concentrations within the 5.0–450.0 and 5–900 ng mL^−1^ range for FVP and LEV, respectively, were prepared by measuring specific volumes of the working stock solutions into 10.0 mL volumetric flasks. Then, the flasks are filled to the mark with distilled water as the diluent. SFS was performed, with Δλ set to 20 nm for FVP and 90 nm for LEV. The scan speed was 6000 nm min^−1^, and the excitation and emission monochromator bandwidths were set to 10 nm. Subsequently, the recorded synchronous fluorescence spectra were utilized to obtain calibration graphs and derived regression equations.

#### Synthetic mixtures analysis

Preparation of synthetic combinations of FVP and LEV in different proportions by transferring different volumes of the corresponding standard solutions (1 μg mL^−1^) into volumetric flasks of 10 mL. After that, each flask was filled with distilled water, thoroughly mixed, and subsequently analyzed following the procedures detailed in the calibration graph construction section. The percentage recoveries can be determined using the relevant regression equations.

#### Application for the determination of FVP and LEV in their tablets

Five tablets of Avipiravir® and five tablets of Tavanic® were obtained from a community pharmacy. Each pharmaceutical preparation of both medications was washed with methanol, an organic solvent used to remove any colored coating. They were thoroughly dried using a clean tissue, then finely ground separately. Specific amounts equivalent to 200 mg of FVP and 500 mg of LEV were weighed and dissolved individually in an appropriate volume of distilled water. The resulting solutions were transferred into 100-mL and 250-mL volumetric flasks for FVP and LEV, respectively.

Subsequently, 70 mL of distilled water was added to each flask, and the solutions were sonicated for 15 min for FVP and 10 min for LEV, respectively. The volumes were then adjusted to the mark with the same solvent and filtered. A 0.1 mL aliquot of the filtrate from each of FVP and LEV was transferred and mixed thoroughly. The mixed filtrate was diluted with distilled water to produce a working standard solution at 2 μg mL^−1^ for each of the two medications. This working standard solution was further diluted by transferring serial volumes into 10 mL volumetric flasks, resulting in five mixture preparations of both pharmaceutical drugs, which yielded concentrations of 5, 10, 150, 250, 350, and 450 ng mL^−1^ for FVP and 5, 150, 250, 350, and 900 ng mL^−1^ for LEV. The procedures described in the “Construction of calibration graphs” section were subsequently performed. The nominal content of the tablets was determined using the appropriate regression equation.

#### Standard addition of FVP and LEV

A single volume of the pharmaceutical working standard solution is distributed into three distinct 10 mL volumetric flasks, each containing a concentration of 5 ng mL^−1^ for the two drugs. These solutions were supplemented with an adequate volume of the pure working standard solution for each drug, mixed thoroughly, and subsequently diluted with distilled water to reach concentrations of 10,400 and 450 ng mL^−1^ for FVP, and 10,300 and 450 ng mL^−1^ for LEV. The average Standard Addition for each drug was calculated using the regression equations.

## Results and discussion

### Synchronous spectrofluorimetric measurements

The most significant characteristic of spectrofluorimetry is its exceptional sensitivity, which makes it a time-saving, easy-to-use, and economical process^57^. FVP and LEV exhibited strong naturally emitted fluorescence signals at 435 nm and 480 nm, respectively, when excited at 350 nm and 300 nm.

**Fig. 1:**
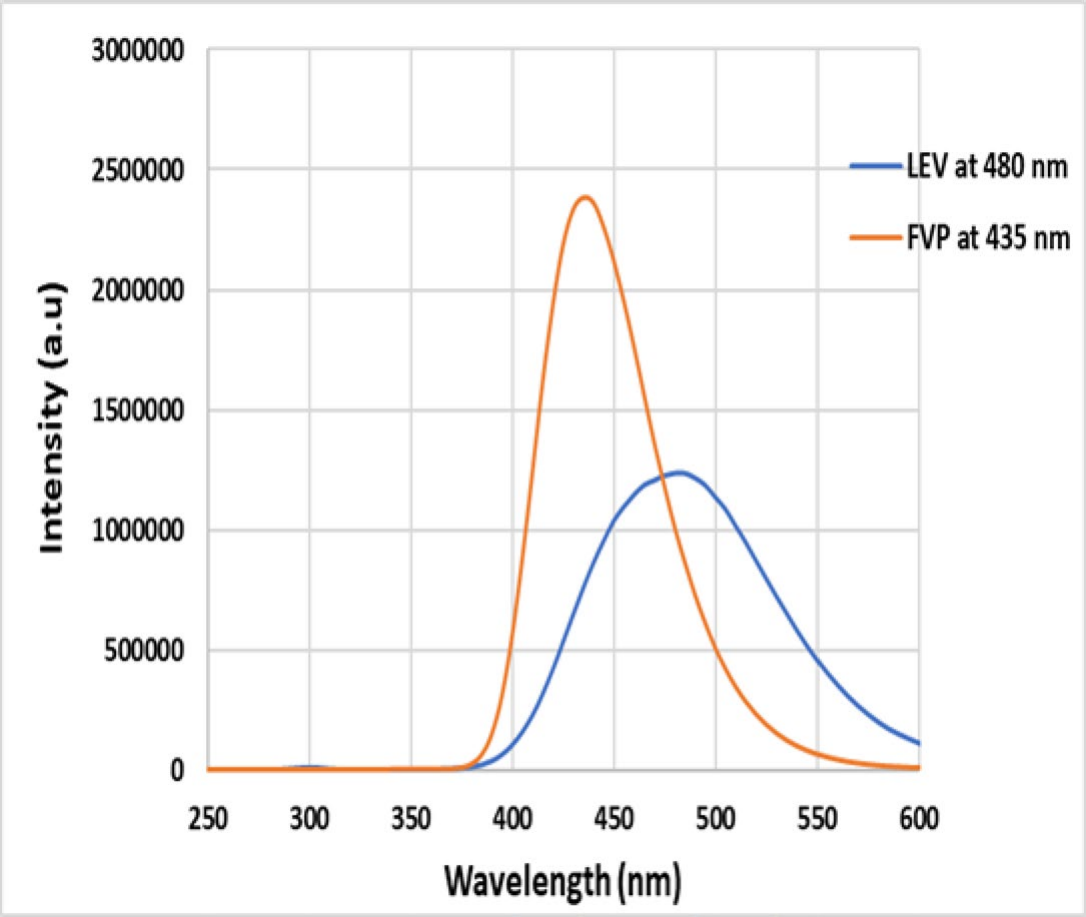
Native Fluorescence spectra of FVP at 435 nm, and LEV at 480 nm.

Figure 1 illustrates the Fluorescence spectra of FVP and LEV at 435 nm and 480 nm, respectively. The overlap between the two spectra is apparent, hindering the simultaneous quantification of these drugs using traditional fluorescence methods. Therefore, we employed Synchronous Spectrofluorimetry (SFS) to determine both drugs concurrently, with their fluorescence spectra distinctly separated. Under the specified experimental conditions, FVP and LEV were successfully differentiated simultaneously using synchronous spectrofluorimetry at 385 nm and 345 nm, respectively, Fig. S2.

### Optimization of experimental conditions

A comprehensive examination and adjustment of several parameters affecting drug fluorescence intensities were conducted. The variables Δλ, pH, and the type of diluting solvent were analyzed.

#### Selection of Δλ

**Fig. 2:**
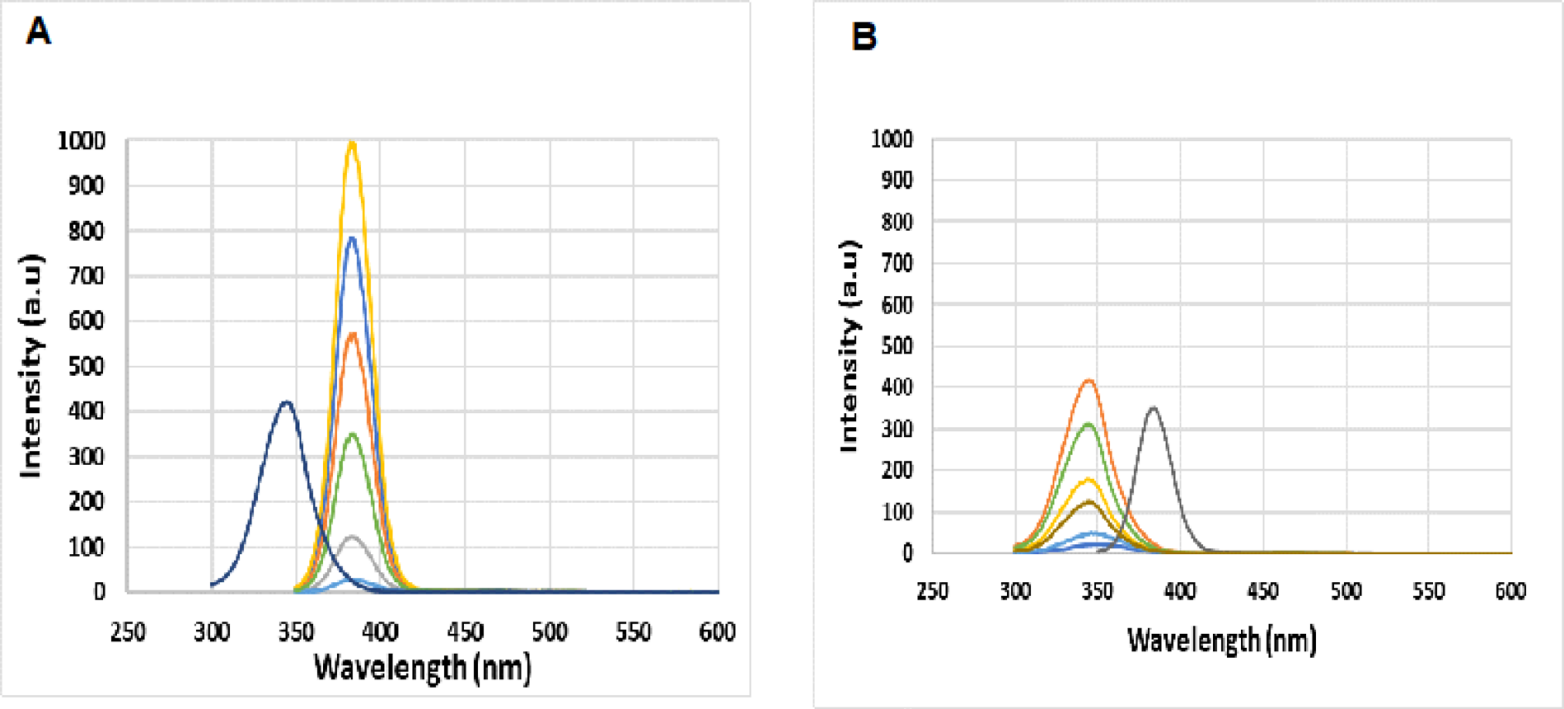
Synchronous fluorescence spectra of: (**a**) FVP (5–450 ng mL^−1^) in the presence of LEV (900 ng mL^−1^) at 385 nm, and (**b**) LEV (5–900 ng mL^−1^) in the presence of FVP (100 ng mL^−1^) at 345 nm.

Simultaneous emission spectra of FVP and LEV were recorded at different Δλ values. It is essential to conduct the SF scanning procedure with optimal sensitivity and resolution, as these factors directly influence bandwidth, spectral structure, and signal strength. Consequently, a wide range of 20–200 nm was examined for both drugs. It was found that the ideal Δλ for FVP and LEV are 20 nm and 90 nm, respectively, regarding excellent resolution, regular peak shapes, and the highest signal levels, Fig. 2. Unreliable fluorescence intensities for both drugs were observed when other values were used instead of the ideal value, as shown in Fig. S3.

#### pH and buffer volume effects

The pH of the diluting medium using borate buffers was examined over the pH range of 4.2 to 8, focusing on its effect on the fluorescence intensity of synchronous spectrofluorimetry of FVP or LEV. The results indicated no significant differences in fluorescence intensity for both drugs across the different pH levels, Fig. S4a. As a result, pH adjustment seemed unnecessary, allowing all experiments to be performed in a neutral medium, further enhancing the developed method’s advantages.

#### Diluting solvent effect

Various solvents, including distilled water, ethanol, methanol, acetonitrile, 0.1 M HCl, and 0.1 M NaOH, were evaluated for their effects on the intensity of the SF spectra. Among the solvents tested, ethanol and distilled water showed the highest SF intensities. Distilled water was the preferred solvent for achieving optimal Whiteness, yielding the highest SF intensities for the two drugs under investigation, Fig. S4b.

### Method validation

In alignment with ICH Q2(R1) guidelines, the validation parameters, including range, linearity, limit of detection (LOD), limit of quantification (LOQ), accuracy, precision, and selectivity, were evaluated^58,59^.

#### Linearity and range

Under the defined experimental conditions, a graph was created to illustrate the relationship between peak amplitude and drug concentration. This relation was achieved by assessing several drug concentrations (n = 6), resulting in the following regression equations:

$$\begin{aligned} y & = 2.1123 \times + 3.8829\quad {\text{for FVP}} \\ y & = 0.4768 \times + 18.28\quad {\text{for LEV}} \\ \end{aligned}$$

Synchronous fluorescence intensity was observed at 385 nm and 345 nm wavelengths for FVP and LEV, respectively. The concentration ranges were determined to be 5–450 ng mL^−1^ and 5–900 ng mL^−1^ for FVP and LEV, respectively. The parameters provided elucidate the regression data analysis presented in Table 1.

**Table 1:** Regression parameters for the assessment of FVP and LEV by the suggested approach.

Parameter	FVP at 385 nm	LEV at 345 nm
Linearity (ng mL^−1^)	5.0–450.0	5.0–900.0
Correlation coefficient (r)	0.9997	0.9998
Slope	2.1123	1232.5
Intercept	3.8829	723,268
LOD (ng mL^−1^)	0.77	1.37
LOQ (ng mL^−1^)	2.33	4.17
Accuracy (%R ± SD)	99.34 ± 1.10	98.78 ± 0.44
Precision (RSD)%	(0.14–0.94)	(0.12–1.19)

#### Detection and quantitation limits

The limit of detection (LOD) was identified as the lowest concentration of the substance that could be reliably detected. The limit of quantification (LOQ) is the lowest concentration that can be accurately measured^58^. The LOD values were 0.77 and 1.37 ng mL^−1^ for FVP and LEV, respectively. The LOQ values were 2.33 and 4.17 ng mL^−1^, respectively. The sensitivities of the selected approach are presented in Table 1.

#### Accuracy and precision

Three different concentrations, each with three duplicates from pure medications, were evaluated to determine the method’s accuracy. The intra- and inter-recovery (%R) were assessed. The intra-recovery was evaluated on the same day as the analysis, while the inter-recovery (%R) was tested over three days, with a minimum interval of 3 days. The values obtained confirmed the method’s high accuracy, as shown in Table S1. The intra-recovery ranges were (99.66–100.67) and (98.87–100.18) for FVP and LEV, respectively. Additionally, the inter-recovery (%R) ranges were (99.28–100.90) and (99.40–100.24) for FVP and LEV, respectively.

As detailed in the accuracy section, the repeatability (intra-precision) and inter-precision of the suggested approach were evaluated by testing three different concentrations, each with three duplicates. The results indicated that the relative standard deviations were minimal, as presented in Table S1. The intra-day precision (%RSD) ranges were (0.15–0.94) for FVP, and (0.12–0.62) for LEV. Furthermore, the interday precision (%RSD) ranges were (0.22–0.65) for FVP and (0.48–1.19) for LEV.

#### Selectivity

The established method facilitates the simultaneous quantification of FVP and LEV in synthetic mixtures. Three lab-combined preparations that included both FVP and LEV were examined. Linear regression equations were used to determine the concentrations of both substances in the synthetic combinations, as shown in Table S2. The results indicate that the method under investigation exhibits high selectivity, demonstrating its ability to consistently and specifically quantify the analyte in the presence of other excipients in the sample medium. Mean recovery% ± SD were 99.40 ± 0.19 and 99.16 ± 1.00 for FVP and LEV, respectively.

The effectiveness of the suggested methods was further evaluated using the standard addition technique; the average recoveries were 99.93% ± 1.19 and 100.15% ± 0.91 for FVP and LEV, respectively, in their tablet dosage form. These findings were statistically compared with those from the reported methods, which use direct UV spectrophotometry for FVP and LEV^28,29^, as shown in Table 2.

**Table 2:** Comparative statistics between the test results gained from the analysis of FVP and LEV using the proposed methodology and the reported ones.

Parameter	Proposed method		Reported methods**	
	FVP	LEV	FVP^35^	LEV^36^
Mean recovery %	98.99	99.47	100.89	99.32
SD	1.16	0.89	1.39	1.04
%RSD	1.17	0.90	1.37	1.04
t-value*	1.592	0.134	–	–
F-value*	4.510	2.164	–	–
Standard addition mean% ± S.D	99.93 ± 1.19	100.15 ± 0.51	–	–

*The tabulated values for the *t*-test and F-test (at 95% confidence interval).

**Ref.^35^ UV spectrophotometric estimation of FVP using an Ethanol: Water (30:70) solvent, with λ max at 231 nm.

**Ref.^36^ UV spectrophotometric determination of LEV using methanol as solvent, *UV detection* at 298 nm.

## Quantification of FVP and LEV in their tablets

The accuracy approach was also evaluated by examining typical excipient interference when measuring the target drugs in their formulations. This was confirmed by comparing the results from the tablet analysis with those from a reference approach within the range of the analytical procedure. The results indicated an accepted recovery with no interference from the excipients or drug-drug interference, Table 2. Statistical analysis^59^ employing the Student t-test and the variance ratio F-test revealed no noticeable differences at 95% confidence interval in precision and accuracy between the two methods, as shown in Table 2. The proposed method is dramatically more sensitive. This suggests its capability to detect trace impurities, perform pharmacokinetic studies, or analyze samples with very low analyte concentrations, unlike the reported UV methods, which are suited for bulk assays.

## Comparison of the proposed method versus the published ones

The proposed synchronous spectrofluorometric method exhibits notable analytical benefits for the concurrent determination of both analytes. A key advantage is the vast linear dynamic range, surpassing those reported in the current literature, thereby broadening applicability across a wide concentration range without the need for sample dilution or pre-concentration. The linear range of 5–450 ng mL^−1^ for FVP significantly exceeds the narrower ranges reported in prior studies, such as 2–13 ng mL^−1^^31^ and 10–100 ng mL^−1^^34^. For LEV, the range of 5–900 ng mL^−1^ offers a significant improvement over previous methods, which were limited to 0.3–18.0 μg mL^−1^^35^ or 10–100 ng mL^−1^^36^ (comparative data are summarized in Tables S3 and S4). The quantitative determination of FVP shows similar or slightly lower sensitivity than methods using fluorescent probes or nanoparticle-enhanced detection^33,37^.

**Fig. 3:**
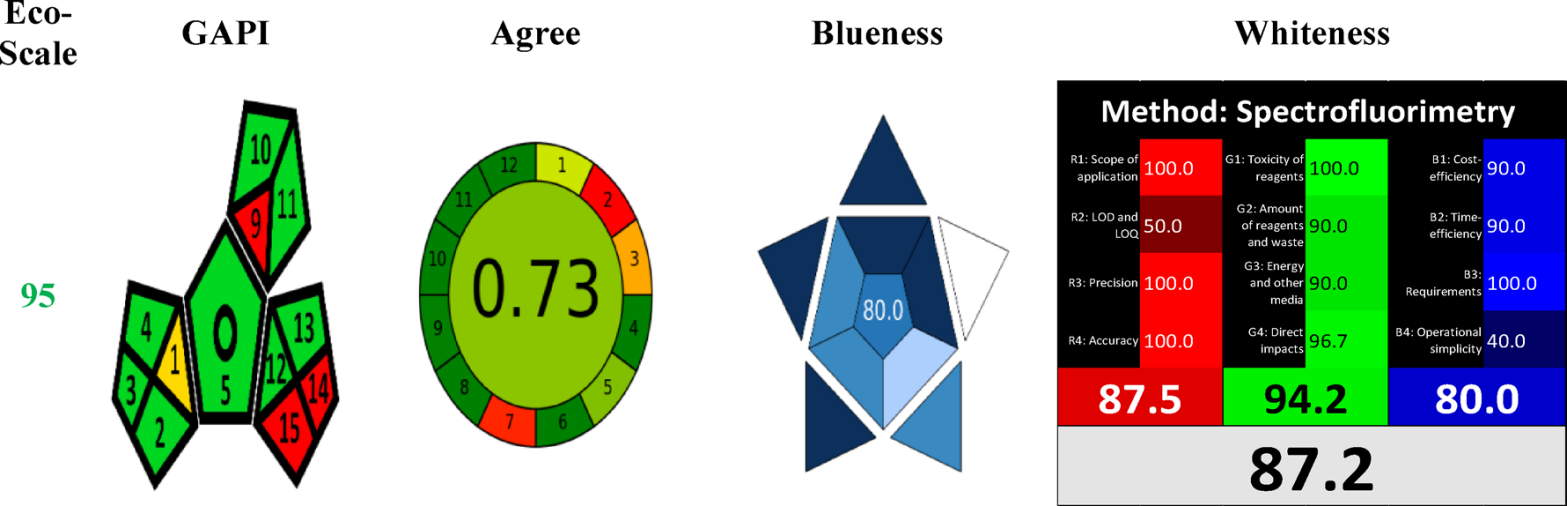
Results of the proposed method’s greenness, blueness, and whiteness assessment.

Additional practical and analytical improvements enhance the method’s utility. The use of distilled water as a solvent offers a cost-effective, environmentally friendly alternative to the organic or buffered solvent systems used in other protocols for LEV determination^37–39^. The suggested method has achieved excellent environmental sustainability and reduced waste, acquiring a high score across all these assessments, as shown in Fig. 3. Greenness is assessed using three tools: Eco-Scale, GAPI, and AGREE. Additionally, blueness and whiteness are evaluated using the BAGI and RGB 12 tools, respectively, while other reported methods lack appraisal of blueness or whiteness evaluation^1,31–39^. A current limitation is its untested performance in complex biological matrices, such as spiked human plasma.

## Greenness assessment of the proposed method

### Green analytical chemistry

Green analytical chemistry aims to develop methods that produce less hazardous waste, are safer for users, and are more environmentally friendly. This objective can be accomplished by creating new analytical techniques. More commonly, it can be achieved by adapting old methods to include processes that utilize either less hazardous chemicals or, when suitable, reduced quantities of dangerous substances^60^.

### Green assessment tools

Three tools are mentioned to evaluate the “greenness” of protocols, each with unique strengths and weaknesses. Ideally, the most effective approach would be to utilize all of them to gather comprehensive information^61^.

#### Eco-scale

The Eco-Scale is a valuable, half-quantitative, innovative, and thorough method for assessing the environmental friendliness of analytical techniques^62^. This approach involves allocating penalty points to aspects of an analytical process that deviate from the principles of optimal green analysis. The Eco-Scale is characterized by the use of cost-effective materials, room-temperature execution, 100% yield, and safety for both the operator and the environment. For every parameter that diverges from this “ideal value,” penalty points are applied, resulting in a reduced overall score. A higher grade indicates that organic preparation is more eco-friendly and cost-effective. The overall penalty points for the proposed method are 5, while the total score is 95, indicating excellent green analysis, Fig. 3.

#### The green analytical procedure index (GAPI)

GAPI assesses the environmental sustainability of an entire analytical process, from sample collection to final analysis. Within GAPI, a unique symbol featuring five pentagrams is employed to evaluate and quantify the environmental impact of each phase of an analytical procedure, with a color-coded system ranging from green to yellow to red, indicating minimum, moderate, and elevated impacts, respectively^63^. Figure 3 shows three red zones due to solvent and waste volumes exceeding 100 mL and the absence of waste treatment. However, these results are not considered a drawback of our suggested method, given the use of distilled water, an excellent green solvent.

#### AGREE metric

The calculations are based on 12 parameters corresponding to the 12 parameters of green analytical chemistry. Each principle or parameter is assigned a score ranging from 0 to 1, reflecting the hazard level associated with its greenness. The AGREE tool illustrates greenness in a traditional clock shape, featuring numbers 1 to 12 around the circumference^64^. The method’s score is 0.73, a value close to 1, confirming method greenness, Fig. 3.

## Blueness assessment of the proposed method

The blue applicability grade index (BAGI) is an innovative tool for evaluating the practicality of analytical chemistry methods within the context of white analytical chemistry (WAC)^65^. In contrast to conventional green metrics that emphasize environmental impact, BAGI focuses on ten essential attributes that are vital to the applicability of methods^66^. The BAGI metric tool yields two forms of results: a pictogram and a numerical score. Figure 3 shows that the method’s score is 80.0, a value close to 1, confirming the technique’s blueness.

## Whiteness assessment of the proposed method

White Analytical Chemistry (WAC) expands on the principles of GAC by incorporating three key areas: efficiency (red principles), environmental safety (green principles), and practical economics (blue principles). The twelve principles of WAC are organized into four green rules (G1–G4), four red principles (R1–R4), and four blue principles (B1–B4)^67^. The method’s score is 87.2, a value approaching 1, thereby affirming its Whiteness, as illustrated in Fig. 3.

## Conclusion

A synchronous fluorescence spectrofluorimetric method has been developed for the first time to simultaneously quantify favipiravir (FVP) and levofloxacin (LEV), two drugs co-administered in the treatment of coronavirus infection, in both bulk and pharmaceutical dosage forms. Fortunately, this technique demonstrates significantly greater sensitivity than conventional UV-spectrophotometric methods, making it particularly suitable for trace-level analysis. The proposed method meets modern needs for high sensitivity and selectivity, making it applicable to challenging areas such as bioanalysis, pharmacokinetic studies, impurity profiling, and the analysis of low-dose formulations. Future research will concentrate on the effects of biological matrix components and potential interferences in spiked human plasma, including the presence of likely co-administered drugs such as paracetamol, oseltamivir, and steroids. Moreover, the new protocol yields better greenness and whiteness scores than other reported methods. These combined advantages support the adoption of this novel approach for routine quality control assessment of both analytes.

## Supplementary Information

Below is the link to the electronic supplementary material.

Supplementary Material 1

## Data Availability

All data generated or analyzed during this study are included in this published article (and its supplementary information file).
