# Supplementary material for: Sustainable dual-drug analysis: a synchronous spectrofluorimetric approach with integrated greenness and whiteness metrics for favipiravir and levofloxacin
Source: Sci Rep. 2026 Feb 3;16:4718. doi: 10.1038/s41598-026-35670-8 (PMC12868843; doi:10.1038/s41598-026-35670-8)
Supplement: Supplementary file 1 — Supplementary Material 1 [file 41598_2026_35670_MOESM1_ESM.pdf]

Supplementary Data

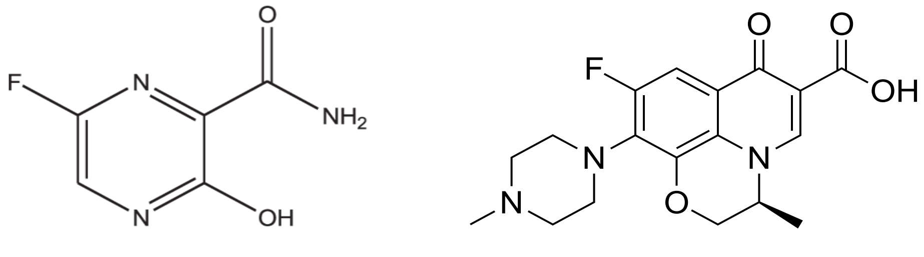

A b

Fig. S1: The chemical structural of (a) FVP and (b) LEV

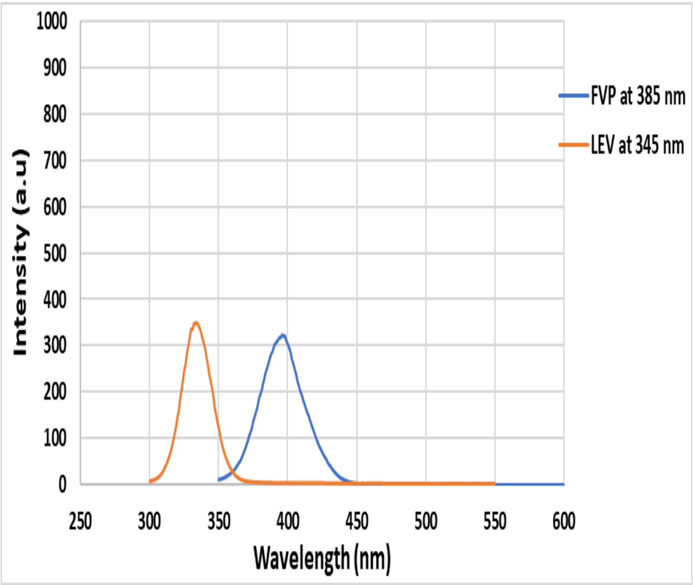

Fig. S2: Synchronous spectra of FVP and LEV at 385 nm (Δλ 20 nm) and 345 nm (Δλ 90 nm).

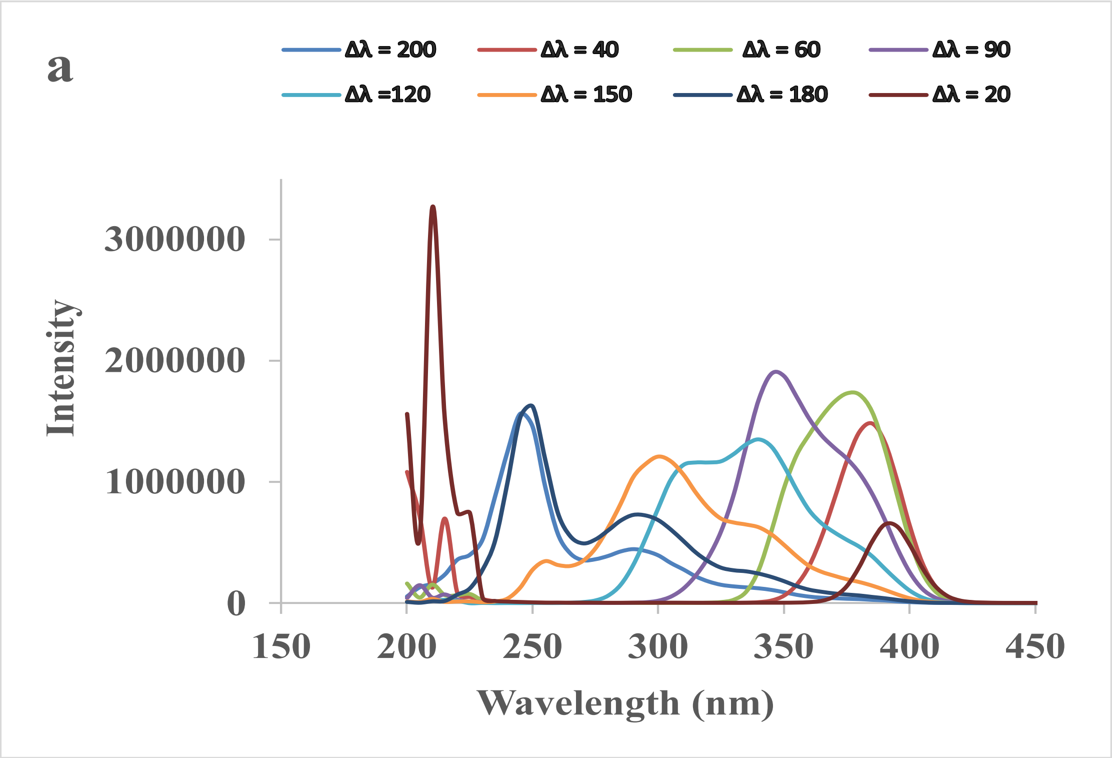

Fig S3a: Effect of selection of Δ λ on synchronous spectra of FVP

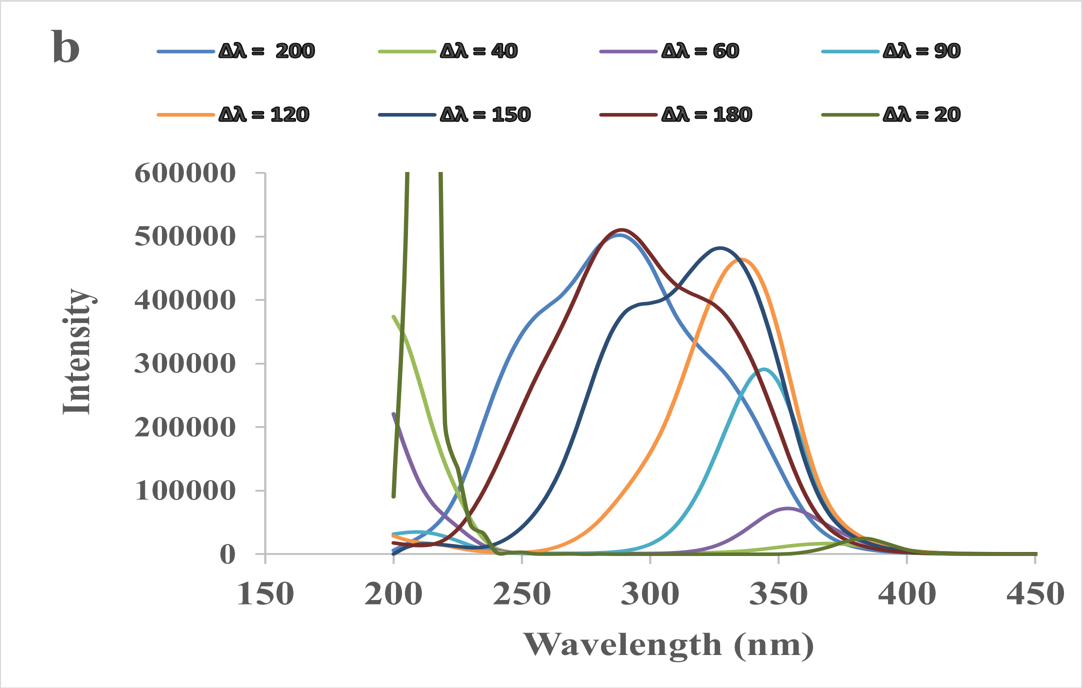

Fig S3b: Effect of selection of Δ λ on synchronous spectra of LEV

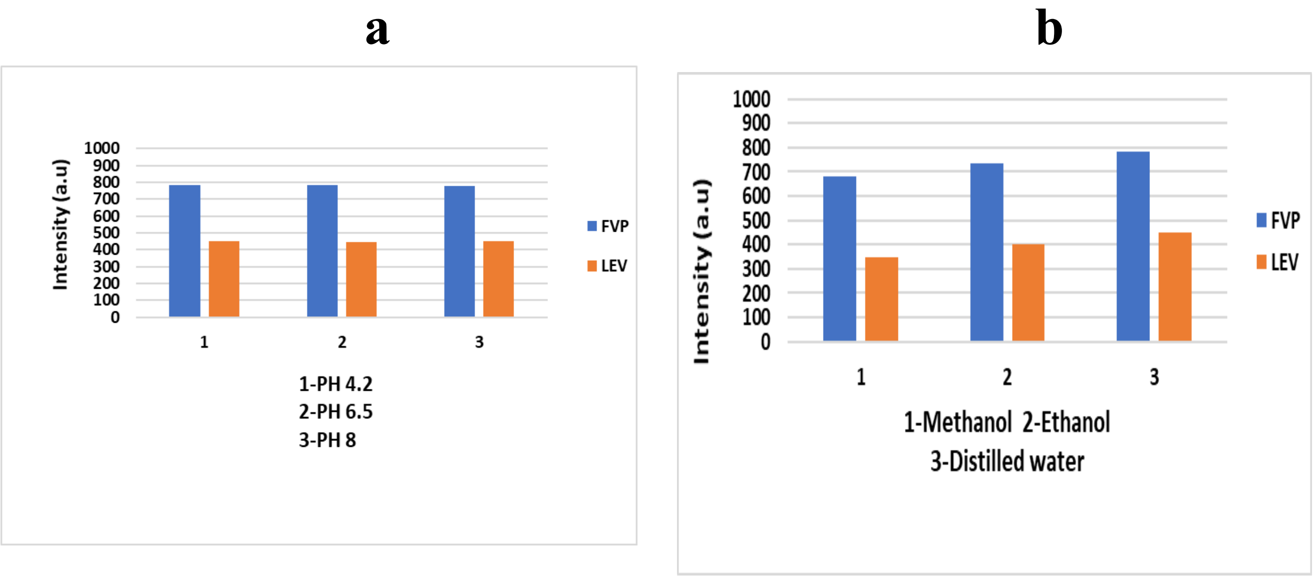

Fig S4: (a) pH, buffer volume and (b) Diluting effect on Synchronous spectra of FVP and LEV

Table S1: Evaluation of accuracy and precision for the quantification of FVP and LEV by the established approach

Drug	Taken (ng mL^{-1})	Intra-day		Inter-day	
		Found ±SD	Precision (%RSD)	Found ±SD	Precision (%RSD)
FVP	5	4.98±0.14	0.15	4.96±0.64	0.65
	200	100.05±0.94	0.94	201.8±0.22	0.22
	450	453±0.38	0.37	452.6±0.44	0.44
LEV	5	4.93±0.62	0.62	4.94±0.18	0.19
	250	250.5±0.54	0.55	250.6±0.62	0.62
	900	99.87±0.12	0.12	894.70±0.48	0.49
*Mean %±SD		100.13 ±0.51		99.53±0.66	

Table S2: Evaluation of the selectivity of the assessment of FVP and LEV in their pure lab combination by the selected methodology

Taken (ng mL^{-1})		FVP	LEV
FVP	LEV	% Recovery	% Recovery
100	100	99.39	100.61
100	200	99.25	98.27
200	100	99.29	98.66
100	250	99.69	99.32
*Mean %±SD		99.41±0.20	99.21±.103

*Mean for three determinations

Table S3: A comparison between the suggested spectrofluorimetry and other published spectrofluorimetric methodologies of FVP determination

Technique	Proposed method	FVP					
		[1]	[31]	[32]	[33]	[34]	
	Synchronous	Native	Synchronous factorized	Native	Native	**Method I** Native	**Method II** Synchronous
λ_{ex} (nm)	350 for FVP	323	364	361	360	323	323
λ_{em} (nm)	385 for FVP	436	430	432	430	432<br>λ of calibration curve = 468.8	432<br>λ of calibration curve = 364
Synchronous Δλ (nm)	Δλ = 20 for FVP	-	Δλ = 50	-	-	-	Δλ = 60
Linearity (ng mL^{-1})	5-450 for FVP	20-350	2–13	48–192 in plasma<br>40–280 in formulation	5.0–200.0	10–100	1-14
Solvent	Distilled water	De-ionized water	Distilled water	Distilled water	De-ionized water	De-ionized water	
Application	Bulk powder and pharmaceutical formulations	Tablet and spiked human plasma	Pharmaceutical, biological, and environmental matrices	Pharmaceutical formulations and spiked human plasma	Tablet and spiked human plasma	Tablet and spiked human plasma	

Table S4: A comparison between the suggested spectrofluorimetry and other published spectrofluorimetric methodologies of LEV determination

	Proposed method	LEV				
		[35]	[36]	[37]	[38]	[39]
Technique	Synchronous	Native (using L-tryptophan as a fluorescent reagent)	Native	Native	Native	Native
λ_{ex} (nm)	300 for LEV	281	330	312	280	290
λ_{em} (nm)	345 for LEV	365	480	553	495	475
Synchronous $\Delta\lambda$ (nm)	$\Delta\lambda$ = 90 for LEV	-	-	-	-	-
Linearity	5-900 ng mL^{-1}	0.3-18.0 µg mL^{-1}	10–100 ng mL^{-1}	0.068–1.448 µg mL^{-1}	0.25–12 µg mL^{-1}	0.0004–0.4 µg mL^{-1}
Solvent	Distilled water	Methanol	Distilled water	Aqueous with 0.1 M NaOH	Methanol / De-ionized Water	Aqueous Acetate-ammonia buffer
Application	Bulk powder and pharmaceutical formulations	Dosage forms	Dosage forms and biological fluids	Pure powder and tablets	Pharmaceutical formulations	Natural water and tablets
